# On the Use and Interpretation of Areola/Nipple Retention as a Biomarker for Anti-androgenic Effects in Rat Toxicity Studies

**DOI:** 10.3389/ftox.2021.730752

**Published:** 2021-10-27

**Authors:** Camilla Lindgren Schwartz, Sofie Christiansen, Ulla Hass, Louise Ramhøj, Marta Axelstad, Nathalie Michelle Löbl, Terje Svingen

**Affiliations:** Division of Diet, Disease Prevention and Toxicology, National Food Institute, Technical University of Denmark, Kongens Lyngby, Denmark

**Keywords:** risk assessment, endocrine disruption, nipple retention, rats, anogenital distance, anti-androgen

## Abstract

Areola/nipple retention (NR) is an established biomarker for an anti-androgenic mode of action in rat toxicity studies. It is a mandatory measurement under several OECD test guidelines and is typically assessed in combination with anogenital distance (AGD). Both NR and AGD are considered retrospective biomarkers of insufficient androgen signaling during the masculinization programming window in male fetuses. However, there are still aspects concerning NR as a biomarker for endocrine disruption that remains to be clarified. For instance, can NR be regarded a permanent adverse effect? Is it a redundant measurement if AGD is assessed in the same study? Is NR equally sensitive and specific to anti-androgenic chemical substances as a shortening of male AGD? In this review we discuss these and other aspects concerning the use of NR as a biomarker in toxicity studies. We have collected available literature from rat toxicity studies that have reported on NR and synthesized the data in order to draw a clearer picture about the sensitivity and specificity of NR as an effect biomarker for an anti-androgenic mode of action, including comparisons to AGD measurements. We carefully conclude that NR and AGD in rats for the most part display similar sensitivity and specificity, but that there are clear exceptions which support the continued assessment of both endpoints in relevant reproductive toxicity studies. Available literature also support the view that NR in infant male rats signifies a high risk for permanent nipples in adulthood. Finally, the literature suggests that the mechanisms of action leading from a chemical stressor event to either NR or short AGD in male offspring are overlapping with respect to canonical androgen signaling, yet differ with respect to other mechanisms of action.

## Introduction

Over the past few decades we have witnessed an increase in male reproductive disorders such as cryptorchidism and hypospadias in new-born boys, or infertility and testis cancers in young men (Diamanti-Kandarakis et al., 2009; Skakkebaek et al., 2016). These reproductive disorders have several known genetic factors, but these cannot alone account for the growing incidence rates (Skakkebaek et al., 2001). Thus, environmental factors must also be involved and developmental exposure to endocrine disrupting chemicals (EDCs) have been suggested to play a significant role in causing reproductive disorders (Diamanti-Kandarakis et al., 2009).

With respect to male reproductive disorders, chemical substances with anti-androgenic properties such as many phthalates and pesticides are of particular concern (Gray 2001; Schwartz et al., 2019a). This is because androgen signaling plays a central role in masculinizing the male fetus and thus blocking androgen action perturbs these virilization events. This failure to become fully masculinized can manifest in different ways. One classical morphometric biomarker of fetal masculinization is the anogenital distance (AGD). Under normal circumstances AGD is approximately twice as long in males as in females and this measure can therefore be used to determine the sex of various animal species, including cats and rodents (Hotchkiss and Vandenbergh 2005), but decreased AGD can also be used as a biomarker indicating that the male fetus is generally under-masculinized (Schwartz et al., 2019a).

Another morphometric biomarker of fetal androgen action is areola/nipple retention, in this review simply referred to as nipple retention (NR). In contrast to humans where both males and females have nipples, common laboratory strains of *Rattus norwegicus* and *Mus musculus* are sexually dimorphic in the number of nipples. Whereas female rats e.g. Wistar and Sprague Dawley normally have 12 nipples (six pairs), the males normally have none (Imperato-McGinley et al., 1986). In common laboratory mice strains the ratio is 10 nipples (five pairs) in females to zero in males (Mayer et al., 2008). Assessment of NR in male rat pups involves the detection of relatively small, dark spots located along the “milk lines” in positions corresponding to where the female nipples are located. In fact, when visually assessed in neonatal offspring–optimally around postnatal day 12/13 – they are only observed as pigmented patches (areolas) rather than actual nipples. If observed under a dissecting microscope the areolae with nipple buds are visible in both infant female and treated male rats. In mice and rats, the nipple anlagen in males regress in response to androgen signaling during development (Kratochwil 1977). Low levels of androgen signaling during this developmental window–as in female fetuses–will allow for the formation of nipples also in the male offspring. NR can thus be used as a retrospective biomarker of fetal androgen action in rats, where the presence of nipples in males signifies insufficient masculinization similarly to a reduced AGD.

In this review, we provide an overview of how various chemical classes can cause NR in male rat offspring and discuss the differences between NR and AGD as biomarkers for anti-androgenicity. We review the current use of NR in regulatory toxicology (focusing solely on rat studies) and provide guidance on how to measure NR to minimize error and bias. Finally, through an extensive literature review of relevant toxicological studies in rats covering numerous chemicals, we address three crucial questions that remain to be answered in order to ensure the optimal use of NR assessment in a regulatory context: *1*) Do different chemical classes have different modes of action when affecting NR and if so, how does this relate to their effects on AGD? *2*) When is NR a transient effect and when is it permanent? *3*) How many retained nipples can you expect in control groups? Our intention is to provide a solid reference source to ensure high quality data when assessing NR and thereby improve chemical risk assessment.

## Androgen-dependent Masculinization in Mammals and the Case of Species-specific Nipple Retention

The development of the male phenotype starts with genetic determination of gonadal sex and subsequent differentiation of the testes in XY fetuses (Svingen and Koopman 2013). Once formed, the fetal Leydig cells initiate steroidogenesis which is required for synthesis of testosterone by the fetal testes. In turn, testosterone can be converted to the more potent androgen dihydrotestosterone (DHT) by the enzyme 5α-reductase in some target tissues (Scott et al., 2009). Both testosterone and DHT bind the androgen receptor (AR) to activate androgen-regulated gene transcription and direct development of male accessory sex organs and the general masculinization of the fetus (Jost 1954; MacLeod et al., 2010; Matsushita et al., 2018; Sharpe 2020). It should also be noted, that although rodents seem to rely solely on androgens synthesized by the fetus, in humans there is a “backdoor pathway” supply of androgens from the placenta (Sharpe 2020).

With respect to testosterone versus DHT, it is worth noting that tissues in close proximity to the developing fetal testis, such as the Wolffian ducts, are exposed to high concentrations of androgens and thus conversion to DHT is not required for masculinization. Conversely, tissues more distant to the testes such as the urogenital sinus (from which the prostate and other urogenital structures differentiate), the perineum, and the nipple anlagen are exposed to lower concentrations of testosterone and thus require DHT conversion for masculinization. Because of this spatial gradient of androgens during development, it is likely that peripheral sites (prostate, perineum and nipple anlagen) are more sensitive to chemicals that act on the AR, as opposed to Wolffian duct derivatives. For example, prenatal exposure to the selective AR antagonist flutamide inhibits prostate differentiation at much lower dose levels than what is required to inhibit seminal vesicle differentiation. Conversely, exposure to phthalates (by reducing testosterone production in the testis) can have a greater effect on Wolffian duct derivate than on more distant tissues such as the prostate (Mylchreest et al., 1999).

**FIGURE 1:**
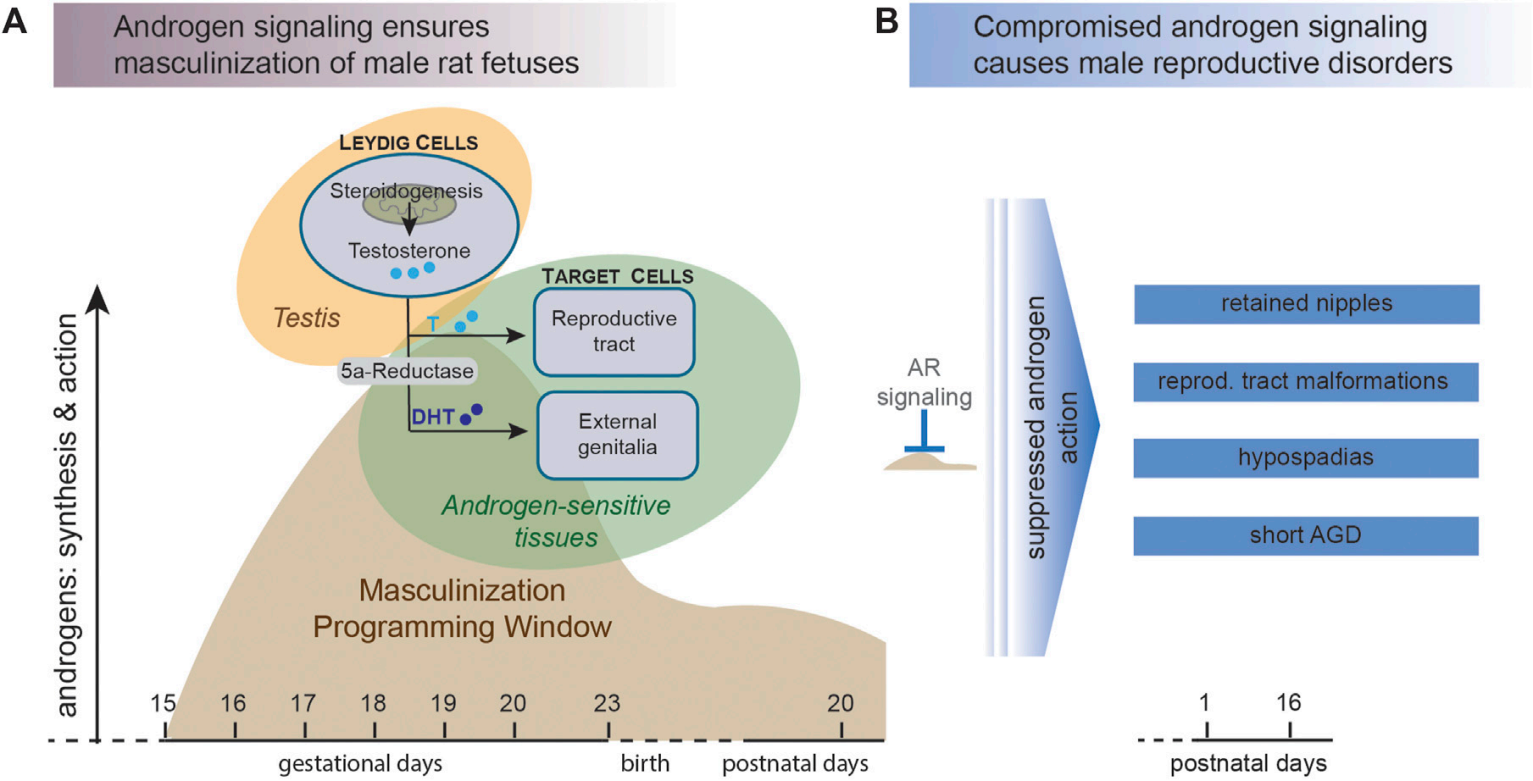
Disrupted androgen action during the male masculinization programming window (MPW) can lead to reproductive disorders in male offspring. **(A)** In mammals, the male fetus is acquiring male characteristics in response to androgen signaling. Testosterone is synthesized by Leydig cells in the fetal testis and then excreted into the circulation. Here, testosterone can be converted to the more potent ligand dihydrotestosterone (DHT), which activates androgen receptor (AR) in cells of the presumptive external genitalia and prompts differentiation into male reproductive tissues. The main masculinization events take place during a specific stage of fetal life denoted the masculinization programming window (MPW), which in rats is around days 15–19 of gestation (adapted from (Welsh et al., 2008). **(B)** Failure to initiate the masculinization differentiation events during the MPW, for instance by exposure to anti-androgenic chemicals, can lead to undervirilization of the male offspring and various reproductive disorders.

In common rats, DHT induces regression of the nipple anlagen (Kratochwil and Schwartz 1976; Kratochwil 1977; Imperato-McGinley et al., 1986). Consequently, male rats do not have nipples. The female rats, who are not exposed to high levels of androgens during the critical stages of development, usually end up having six pairs of nipples, 12 in total. These androgen mediated effects take place during a short window of fetal development (embryonic day 15.5–18.5 in rats), often referred to as the masculinization programming window (MPW) (Welsh et al., 2008; van den Driesche et al., 2012, 2017; Sharpe 2020), and depicted in Figure 1A. Androgen action during the MPW is believed to programme the male reproductive organs in both humans and rats (Welsh et al., 2008; MacLeod et al., 2010), but compared to the rodents the human MPW occurs earlier during fetal life, more precisely in the first trimester between gestation weeks 8–14 (Welsh et al., 2008; Dean and Sharpe 2013). In mammals, disruption of androgen action during the MPW perturbs normal development of the androgen dependent tissues and may manifest as reproductive disorders (Welsh et al., 2008; Sharpe and Skakkebaek 2008; van den Driesche et al., 2017), for instance NR, hypospadias and short AGD (Figure 1B). Although the androgen signaling pathway can be disrupted in different ways, from reduced testosterone synthesis to blocking of AR binding, we refer to compounds that interfere with any step of the androgen signaling pathway as anti-androgenic compounds.

Perturbed androgen signaling during the MPW can result in NR in male rat offspring (Kratochwil 1977; Carruthers and Foster 2005) and is–similarly to AGD–considered a general biomarker of incomplete masculinization (Schwartz et al., 2019a). NR is associated with adverse effects such as hypospadias and cryptorchidism, decreased penile length and reduced seminal vesicle weight in rats (Bowman et al., 2003; Christiansen et al., 2008). Since this sexually dimorphic regression of the male nipples is occurring in rats, but not in humans, NR is not a useful biomarker in human epidemiological studies. That said, the phenomenon of nipple regression is naturally taking place in humans, albeit in both sexes (*see* section 8). Nevertheless, even though assessment of NR is not directly transferable to the human situation, it is a clear readout of insufficient androgen signaling during the MPW in rats. And since disrupted androgen signaling is directly relevant for adverse male reproductive health effects in humans, NR is a relevant biomarker which can be used to identify anti-androgenic chemicals and therefore an important endpoint to assess in rat toxicological studies that aim to predict endocrine disruption and adverse effects on human health.

## Nipple Retention in Rat Toxicity Studies

### A Compilation of Literature Reporting on NR in Rat Toxicity Studies

To provide an overview of available open-literature data pertaining to NR in toxicity studies, we carried out a search in PubMed (https://pubmed.ncbi.nlm.nih.gov/), as well as reassessing our extensive study compilations from our previous review on AGD (Schwartz et al., 2019a). Our new tables contain all of the information relevant for NR, including occurrence in control animals/groups and sensitivity of the endpoints by elucidating Lowest Observed Adverse Effect Level (LOAEL) of individual studies. Our literature searches were updated using the search strings “nipple AND retention” and “anogenital AND distance” (published until 10.02.21). We included studies if they *1*) contained data on NR *2*) were performed in rats and *3*) reported information on single substance exposures (i.e. not studies on chemical mixtures). The full set of studies is listed in Supplementary Table S1.

We assessed the quality and strength of the results of all studies using a simple weight of evidence (WoE) approach. This included both positive and negative results so that we could draw an overall conclusion for each chemical (Table 1). The level of evidence was denoted “strong,” “moderate,” “weak” or “inconclusive” based on the following criteria. *Strong*: several studies indicating clear and coherent evidence in the absence of conflicts; *Moderate*: one or more studies showing coherent evidence; *Weak*: one or more studies showing clear trend or indication of evidence but not enough available data; *Inconclusive*: studies showing conflicting results or methodological limitations hindering evidence assessment.

**TABLE 1:** Weight of Evidence (WoE) for Nipple Retention (NR) effect patterns.

Substance	NR effects in infant male rats			Permanent nipples	AGD effects	Suggested main ED MOA(s) for NR	References
	Pattern of effect	Comments	Strength of evidence				
Clear dose related effect on both NR and AGD							
Vinclozolin	Clear dose response with maximal effects at 12 nipples	-	Strong	Strong evidence	Clear dose response, maximal decrease of 40–50% (almost to female levels)	Potent AR antagonist	11, 17, 20, 23, 28–29, 45, 60, 67, 78, 80
Procymidone	Clear dose response with maximal effects at 12 nipples	-	Strong	Strong evidence	Clear dose response, maximal decrease of 40% (almost to female levels)	Potent AR antagonist	27, 28, 61, 77
Flutamide	Clear dose response with maximal effects at 12 nipples	-	Strong	Strong evidence	Clear dose response, maximal decrease around 50% (as female levels)	Potent AR antagonist	21–22, 27, 33, 36, 48, 53, 55, 69, 82
Pyrifluquinazon	Dose-response with maximal NR around 9–10 or 100% (all exposed showed NR)	Percent animals with any nipples is more sensitive than AGD, no clear threshold	Strong	Not studied	Dose-response with maximal decrease of 33%	Weak AR antagonist	26
DEHP	Dose-response with maximal NR around 10 or 100%	Maximal NR may be limited by maternal toxicity	Strong	Strong evidence	Dose-response with maximal decrease of 34%	Decreased testosterone	1, 11–12, 24–25, 31, 34, 44, 54, 66, 77
DBP	Dose-response with maximal NR around 10 or 100%	Maximal NR may be limited by maternal toxicity	Strong	Strong evidence	Dose-response with maximal decrease of 25%	Decreased testosterone	4, 9, 16, 31, 36, 39, 44, 55–56, 65, 70, 77
BPP	Dose-response with maximal NR around 5 or 70%	Maximal NR may be limited by maternal toxicity	Strong	Strong evidence	Dose-response with maximal decrease of 30%	Decreased testosterone	24, 30, 75
DiBP	Dose-response with maximal NR around 2 or 74%	One well-performed study	Strong (based on read-across to DBP)	Strong evidence	Dose-response with maximal decrease of 22%	Decreased testosterone	65
Dicyclohexyl phthalate	Maximal NR around 2.7 or 68%	One study, only effect at highest dose	Moderate	Not studied	Maximal decrease of 15%	Decreased testosterone	65
DIHP	Maximal NR around 6.3	One study, only effect at highest dose	Moderate	Not studied	Maximal decrease of 15%	Decreased testosterone	1
DINP	Maximal NR around 3.2 or 22%	Maximal NR may be limited by maternal toxicity	Moderate	Weak evidence in one study	Maximal decrease around 6%	Decreased testosterone	6, 16, 24
DnHP	Dose-response with maximal NR around 81%	One well-performed study	Strong	Strong evidence	Dose-response with maximal decrease of 18%	Decreased testosterone	66
Linuron	Dose-response with maximal NR around 3.7 or 44%	Several studies with consistent results. One negative study had limited group sizes (litters per group)	Strong	Strong evidence	Dose-response with maximal decrease of 31%	AR antagonist, inhibit testosterone synthesis	30, 47, 49, 69, 77
DDE (p,p'-DDE)	Dose-response with maximal NR around 4.8 or 71%	Several studies finding effects. One negative study at similar dose levels report no effect, but data are not shown	Moderate	Moderate evidence (data not shown)	Dose-response with maximal decrease of 14% (conflicting data)	(Potent) AR antagonist	41, 77, 81–82
Shallow dose response curves with effects on both NR or AGD							
Finasteride	Clear dose response with maximal effects at 8–12 nipples or 100%	Very shallow dose-response over a 10,000-fold dosage range	Strong	Moderate evidence	Dose-response with maximal decrease of 38%	5-alfa-reductase inhibition	8, 11, 14–15, 43
Clear effects on NR and no/minor effect on AGD							
Prochloraz	Dose response with maximal effects at two to five nipples or, nipples in 80–90% of males	Examined in several studies	Strong	Moderate evidence	Only seen effect on male AGD in one study (Laier et al. (2006), whereas 6 studies are negative	Inhibiting CYP 19 enzyme activities, inhibit steroidogenesis and antagonize the AR *in vitro*	11, 28, 37, 52, 58, 76
Tebuconazole	Dose-response with maximal NR around 3.1	Two well-performed studies, but data not clearly consistent	Moderate	Not studied	No effect	Disruption of steroidogenesis including inhibition of CYP19, and AR antagonism	28, 72
Epoxiconazole	Dose-response with maximal NR around 3.4	Two well-performed studies, but data not clearly consistent	Moderate	Not studied	No clear effect (or slightly increased)	Inhibiting CYP enzyme activities	28, 72
Paracetamol	NR of 30%	One well-performed study, but only one dose level	Moderate	Not studied	No effect	Inhibitor of prostaglandin synthesis	3
Nitrotriazolone (1,2,4-triazol-5-one; NTO)	Dose-response with maximal NR of 1.0 or 30%	One well-performed study (OECD TG 443). Very shallow dose-response from 144 to 3.600 mg/L drinking water	Strong	Not studied	No effect	non-receptor mediated modes of action, including effects on Sertoli and Leydig cells, altered steroidogenesis, and/or altered local metabolism of testosterone to dihydrotestosterone (DHT)	40
No/minor effect on NR and effects on AGD							
Bisphenol A (BPA)	Dose response with maximal effects at 0.4 nipples, only statistically significant at 50 mg/kg per day	Only seen in one well-performed study but not in several other studies	Weak	Not found	Christiansen et al., 2014: Dose-response with maximal decrease of 7%, the dose–response curve was very shallow. No clear increase in response was seen with increasing dose. Not found in the other studies	Estrogenic, 5-alfa-reductase inhibition, Weak AR	13, 18–19, 32, 74
Butylparaben	No effect	One well-performed study	Moderate for lack of NR	Not studied	Dose-response with maximal decrease of 7%, the dose–response curve was very shallow. No clear increase in response was seen with increasing dose	Estrogenic mode of action	7
DES	Dose response (shallow) with maximal effects at 0.3–0.5 nipples, effect at all doses	Only seen in one well-performed study (not examined in other studies)	Weak	Not studied	only the low dose of DES (DES-0.003) significantly reduced male AGD index	Estrogenic mode of action	35
Miscellaneous effects							
Ketoconazole	Dose response (shallow) with maximal effects at one nipple, effect at all doses	Found in one well-performed study (not observed in Wolf et al. . 1999)	Weak	Not found	AGD index was shorter in all males exposed to KTZ (2–3%)	inhibiting CYP enzymes and interfering with both androgen and estrogen synthesis	35, 77
Simvastatin	Dose-response with maximal NR around 1.2 or 40%	Found in one study with few litters and pup mortality	Weak	Not found, but inconclusive evidence	Maximal decrease of 10%	Lowering of cholesterol leading to lower T	5
Fenitrothion	Dose-response with maximal NR around 4.1	Only at high dose causing pup mortality	Weak	Not found, but inconclusive evidence	Maximal decrease of 16%	Weak AR antagonist	59, 73
Mancozeb	Dose-response with maximal NR of 0.6	One well-performed study, but very small effect	Weak	Not studied	No effect	No AR antagonism or effect on T *in vitro*	28
PCB 126	No effect	Results not reported	Inconclusive	Not studied	Marginally decreased in high dose group	-	64
Perfluorohexane sulfonate (PFHxS)	Weakly, but significantly, increased NR in trend analysis	One well-performed study, but very small effect	Inconclusive	Not studied	No effect	-	63
Fludioxonil	No effect	One well-performed study, few litters	Inconclusive	Not studied	only the mid dose (and not the high dose) significantly reduced male AGD index	inhibited testosterone synthesis and androstenedione	68
Cyprodinil	No effect	One well-performed study, few litters	Inconclusive	Not studied	only the mid dose (and not the high dose) significantly reduced male AGD index	inhibited testosterone synthesis and androstenedione	68
Dimethomorph	No effect in 1st study, 2nd study showed increased NR at low and high dose, but not at two mid doses	Two studies, conflicting data	Inconclusive	Not studied	1st study: low and mid dose, but not the high dose, significantly reduced male AGD index 2nd study: two highest doses, but not the low and mid dose, significantly reduced male AGD index	inhibited testosterone synthesis and androstenedione	68
No effect on NR and AGD, only one study of each substance							
2-hydroxy-4-methoxybenzophenone (HMB)	No effect	One well-performed study	-	Not studied	No effect	Estrogenic *in vitro* and *in vivo*	57
Acrylamide	No effect	One well-performed study	-	Not studied	No effect	Testicular toxicity, unknown MOA	71
BPC (Bisphenol C)	No effect	One well-performed study	-	Not studied	No effect	Bind AR with high affinity and act as an AR antagonist *in vitro*	26
DEP	No effect	One well-performed study	Moderate for lack of NR	Not found	No effect	Expected negative based on chemical structure	24
DMP	No effect	One well-performed study	Moderate for lack of NR	Not found	No effect	Expected negative based on chemical structure	24
DOTP	No effect	One well-performed study	Moderate for lack of NR	Not found	No effect	Expected negative based on chemical structure	24
Chlozolinate	No effect	Only one study	-	Not found	No effect	Dicarboximide fungicide like the AR-antagonists vinclozolin and procymidone	77
Heptachlor	No effect	Only one study	-	Not studied	No effect	No suggested ED MOA	38
Iprodione	No effect	Only one study	-	No effect	No effect	Dicarboximide fungicide like the AR-antagonists vinclozolin and procymidone	77
Isobornyl acetate	No effect	Only one study	-	Not studied	No effect	No suggested ED MOA	62
Lindane	No effect	Only one study	-	Not studied	No effect	Estrogenic or anti-estrogenic *in vitro*	46
Loratadine	No effect	Only one study	-	Not studied	No effect	No suggested ED MOA	50
OMC	No effect	Only one study	-	Not studied	No effect	Estrogenic *in vitro* and in fish	2
Dienestrol	No effect	Only one study	-	Not studied	No effect	Estrogenic, a catabolic product of diethylstilbestrol (DES)	69
Genistein	No effect	Only one study	-	Not studied	No effect	Estrogenic (phytoestrogen)	10
PCB 169	No effect	Only one study	-	Not studied	No effect	Aryl hydrocarbon Ah receptor	77
EE2 (ethinyl estradiol)	No effect	Examined in several studies	Moderate for lack of NR	Not studied	No effect	Potent, estrogenic mode of action	18–19, 32, 42
Testosterone propionate	No effect	Androgenic effects in female pups	-	Not found	No effect	Androgenic	79–80

The strength of evidence was denoted “strong” “moderate,” “weak” or “inconclusive” based on the following criteria. *Strong*: several studies indicating clear and coherent evidence in the absence of conflicts; *Moderate*: one or more studies showing coherent evidence; *Weak*: one or more studies showing clear trend or indication of evidence but not enough available data; *Inconclusive*: studies showing conflicting results or methodological limitations hindering evidence assessment. DDE, DDT metabolite, dichlorodiphenyl- dichloroethylene; HBM, 2-Hydroxy-4-Methoxybenzone; OMC, Octyl Methoxycinnamate; DMP, dimethyl phthalate; DEP, diethyl phthalate; DBP, dibutyl phthalate; MBuP, monobutyl phthalate; DiBP, di-isobutyl phthalate; DEHP, diethylhexyl phthalate; DHP, di-n-hexyl phthalate; DCHP, dicyclohexyl phthalate; BBP, benzyl butyl phthalate; DnHP, di-n-hexyl phthalate; DHPP, di-n-heptyl phthalate; DiHP, di-isoheptyl phthalate; DnOP, di-n-octyl phthalate; DOTP, dioctyl terephthalate; DiNP, di-isononyl phthalate; DUDP, diundecyl phthalate; DTDP, ditridecyl phthalate; DES, diethylstilbestrol. 1: Andrade et al. (2006), 2: Axelstad et al. (2011), 3: Axelstad et al. (2014), 4: Barlow et al. (2004), 5: Beverly et al. (2019), 6: Boberg et al. (2011), 7: Boberg et al. (2016), 8: Bowman et al. (2003), 9: Carruthers and Foster (2005), 10: Casanova et al. (1999), 11: Christiansen et al. (2009), 12: Christiansen et al. (2010), 13: Christiansen et al. (2014), 14: Clark et al. (1990), 15: Clark et al. (1993), 16: Clewell et al. (2013), 17: Colbert et al. (2005), 18: Delclos et al. (2014), 19: Ferguson et al. (2011), 20: Flick et al. (2017), 21: Foster and Harris (2005), 22: Fussell et al. (2015), 23: Gray (2001), 24: Gray et al. (2000), 25: Gray et al. (2009), 26: Gray et al. (2019), 27: Hass et al. (2007), 28: Hass et al. (2012), 29: Hellwig et al. (2000), 30: Hotchkiss et al. (2004), 31: Howdeshell et al. (2007), 32: Howdeshell et al. (2008), 33: Imperato-McGinley et al. (1992), 34: Jarfelt et al. (2005), 35: Johansson et al. (2021), 36: Kim et al. (2010), 37: Laier et al. (2006), 38: Lawson and Luderer (2004), 39: Lee et al. (2004), 40: Lent et al. (2016), 41: Loeffler and Peterson (1999), 42: Mandrup et al. (2013), 43: Martínez et al. (2011), 44: Martino-Andrade et al. (2009), 45: Matsuura et al. (2005a), 46: Matsuura et al. (2005b), 47: McIntyre et al. (2000), 48: McIntyre et al. (2001), 49: McIntyre et al. (2002), 50: McIntyre et al. (2003), 51: McKee et al. (2006), 52: Melching-Kollmuss et al. (2017), 53: Miyata et al. (2002), 54: Moore et al. (2001), 55: Mylchreest et al. (1999), 56: Mylchreest et al. (2000), 57: Nakamura et al. (2015), 58: Noriega et al. (2005), 59: Okahashi et al. (2005), 60: Ostby et al. (1999a), 61: Ostby et al. (1999b), 62: Politano et al. (2017), 63: Ramhøj et al. (2018), 64: Rice (1999), 65: Saillenfait et al. (2008), 66: Saillenfait et al. (2009), 67: Schneider et al. (2011), 68: Scholze et al. (2020), 69: Schreiber et al. (2020), 70: Souza et al. (2019), 71: Souza et al. (2020), 72: Taxvig et al. (2007), 73: Turner et al. (2002), 74: Tyl et al. (2002), 75: Tyl et al. (2004), 76: Vinggaard et al. (2005), 77: Wolf et al. (1999), 78: Wolf et al. (2000), 79: Wolf et al. (2004), 80: Wolf et al. (2004), 81: Yamasaki et al. (2009), 82: You et al. (1998).

### How Is NR Affected by Different Chemicals?

Based on our literature review we identified 147 relevant reproductive toxicity studies in rats that have reported on the effects of various chemicals on NR in male offspring, as well as co-reporting of AGD (Supplementary Table S1). The following discussion focuses on the results from Table 1, which shows our WoE assessment of effects of each of the listed chemicals. We discuss the various chemical substances with focus on putative or suggested modes of action and how this relates to the observed adverse outcomes. An additional question is whether specific chemical classes cause different effect patterns on NR and AGD. Our aim was to examine if we, based on the collected information, could gain more insight into how chemicals induce these effects and use the information to elaborate more robust modes of action or Adverse Outcome Pathway frameworks (Villeneuve et al., 2014) to be used for chemical safety evaluations.

#### Compounds With a Clear Dose Related Effect on Both NR and AGD

Flutamide, vinclozolin and procymidone all have AR antagonistic activity, and all three show very marked anti-androgenic effects *in vivo*. Flutamide is a drug used for various medical treatments, not least prostate cancers (Miyata et al., 2002). *In vitro,* flutamide antagonizes the AR by preventing receptor binding of both DHT and testosterone (Simard et al., 1986). With regard to NR, studies report a clear dose-response relationship between exposure concentrations and the number of nipples (for all 14 identified flutamide references, *see* Table 1). Three robust studies report on a full dose-response relationship of flutamide, and the retention of 12 nipples in male offspring at doses between 16 and 50 mg/kg bw/day administered during the MPW (McIntyre et al., 2001; Hass et al., 2007; Schreiber et al., 2020). At around 2 weeks of age, these male offspring were practically indistinguishable from their female littermates as regards the presence of nipples. Another study on flutamide exposure in the same dose range (40 mg/kg bw/day) reported only six retained nipples in males (You et al., 1998). It is unclear why only six retained nipples were observed in this study, but regardless there is no doubt that flutamide exposure results in clear effects on NR in rats.

Exposure to the fungicides vinclozolin and procymidone also give rise to dose-dependent NR in male rat offspring (*see* Table 1). Both chemicals display AR antagonism *in vitro* (Kelce et al., 1994; Nellemann et al., 2003; Kleinstreuer et al., 2017; Scholze et al., 2020) and give strong effects on NR *in vivo*. Vinclozolin induces NR with a clear dose-response relationship and, like flutamide, can induce retention of all 12 nipples in prenatally exposed males. This response is achieved at doses around 200 mg/kg bw/day (Wolf et al., 2004; Hass et al., 2007), whereas statistically significant effects on NR start occurring from around 5–10 mg/kg bw/day (Ostby et al., 1999a; Hass et al., 2007). Likewise, procymidone causes a clear dose-response for NR (Hass et al., 2007, 2012) and induces retention of all 12 nipples at a dose of 150 mg/kg (Hass et al., 2007).

Flutamide, vinclozolin and procymidone also induce shorter AGD in male rat offspring and in several studies male AGD decreased to (almost) resemble female AGD (Gray et al., 1994; You et al., 1998; Hellwig et al., 2000; McIntyre et al., 2001; Wolf et al., 2004; Hass et al., 2007). As discussed in more detail in section 5, NR and shortening of AGD in male offspring start manifesting at similar dose levels.

Certain phthalates can induce marked anti-androgenic effects *in vivo* (Table 1). However, phthalates are not as potent in inducing NR as potent AR antagonists and the 5-alpha reductase inhibitors. Instead, phthalates are typically considered to act as anti-androgens by reducing fetal testosterone levels (or by inhibiting testosterone biosynthesis) as this effect is measurable *in vivo.* Thus, it can be potentially illuminating to scrutinize the effect patterns of NR and AGD relative to mechanisms of action and to see if specific modalities are more potent at inducing adverse outcomes than others.

A thorough analysis of the available literature (Table 1 and Supplementary Table S1) reveals that a large percentage of the male pups present with NR within the single studies, but individual male pups rarely have a high number of retained nipples. There are exceptions, however, with diethylhexyl phthalate (DEHP) and dibutyl phthalate (DBP) inducing a high number of retained nipples at high exposure doses. A few studies on DEHP report up to 8–10 retained nipples in individual pups at doses of 750–1000 mg/kg DEHP (Wolf et al., 1999; Moore et al., 2001), even though the majority of DEHP studies find a much lower mean number of retained nipples (Jarfelt et al., 2005; Howdeshell et al., 2007; Gray et al., 2009). A high dose of 500 mg/kg DBP has been reported to induce up to 10 retained nipples in a single study (Barlow et al., 2004), but as with DEHP, the majority of studies report between 1 and 5 retained nipples even at high doses (Wolf et al., 1999; Howdeshell et al., 2007; Martino-Andrade et al., 2009). This effect pattern–low number of retained nipples in individual pups–is also evident for other anti-androgenic phthalates such as benzyl butyl phthalate (BBP) (Hotchkiss et al., 2004; Tyl et al., 2004), di-isononyl phthalate (DiNP) (Boberg et al., 2011), di-isobutyl phthalate (DiBP) (Saillenfait et al., 2008), Dicyclohexyl phthalate (DCHP) (Yamasaki et al., 2009), di-isoheptyl phthalate (DiHP) (McKee et al., 2006) and di-n-hexyl phthalate (DnHP) (Saillenfait et al., 2009). However, overall the phthalates may appear to cause less marked effects on both NR and AGD than the above mentioned chemicals, because the doses eliciting a very potent anti-androgenic response cannot be administered due to maternal toxicity. An additional explanation for the less marked effects of phthalates on AGD and nipple retention is that the phthalates disrupt androgen-regulated male sexual differentiation without interacting directly with the AR, as does e.g. flutamide (Mylchreest et al., 1999).

The above-mentioned phthalates typically induce shorter male AGD in the range of 6–34% reductions compared to control at exposure concentrations similar to those inducing NR. In other words, anti-androgenic phthalates seem to elicit effects on NR and AGD at comparable doses (Table 1).

Pyrifluquinazon (PFQ), a new active ingredient insecticide, has been tested in one study investigating its effects both *in vitro* and *in vivo* (Gray et al., 2019). PFQ showed weak AR antagonistic effects *in vitro* but surprisingly this compound elicited a very potent NR response *in vivo* with maximal NR around 9–10. This response was seen at a dose of 100 mg/day, a dose which caused a non-significant reduced maternal weight gain during dosing. PFQ had a dose-dependent but less marked effect on AGD which was maximally decreased by 33%.

Linuron is a well-characterized anti-androgenic herbicide that can antagonize the AR *in vitro*, but also inhibit the synthesis of androgens such as testosterone and androstenedione (Scholze et al., 2020). The performed *in vivo* studies show decreased testosterone levels indicating that this compound could also affect steroidogenesis *in vivo*. The maximally observed number of nipples in linuron exposed male offspring was 3.7 at 50 mg/kg (McIntyre et al., 2000). The effect on NR was much weaker than for PFQ (Gray et al., 2019), whereas the maximum effect on AGD (31% shorter) was comparable to PFQ. The dose levels of linuron that caused the maximum effects on NR and AGD also caused a marked degree of maternal and developmental toxicity, which prevented higher doses from being tested.

Dichlorodiphenyldichloroethylene (p,p’-DDE) is the main metabolite of the insecticide DDT and is an AR antagonist that inhibits AR-dependent gene expression *in vitro* (Kelce et al., 1995, 1997). This insecticide metabolite has been examined in several *in vivo* rat studies, with somewhat conflicting results. DDE induced a maximum NR around 4.8 (Loeffler and Peterson 1999), whereas a relatively small maximum effect on AGD of 14% was reported in one of the studies (You et al., 1998).

Together, the results from these AR antagonists indicate that *in vitro* knowledge alone is not sufficient to predict the *in vivo* potency on NR and AGD. This can probably be explained by the different toxicokinetic properties of these AR antagonists *in vivo*; important data that could increase the predictive power of *in vitro* data in the future.

#### Finasteride Has Shallow Dose Response Curves With Effects on Both NR and AGD

Finasteride, like flutamide, is an anti-androgenic drug used for prostate cancer treatment (FDA 2011). Rather than antagonizing the AR, however, finasteride works by inhibiting the enzyme 5α-reductase, thereby blocking the conversion of testosterone to DHT (Clark et al., 1990). Since DHT is the main androgen responsible for inducing regression of nipples in male laboratory rats, it is not surprising that finasteride exposure can induce NR. In fact, finasteride has been shown to be very potent *in vivo*, at very low doses of only 0.01 mg/kg bw/day (Bowman et al., 2003; Christiansen et al., 2009) resulting in statistically significant NR, while doses from 0.1 and up to 100 mg/kg result in 8–12 nipples in male offspring (Bowman et al., 2003; Christiansen et al., 2009; Martínez et al., 2011). Finasteride also has a potent effect on male AGD in rats, but notably, maximal decreases were 38% (Clark et al., 1990) and thus not as marked as the 50% observed for the AR-antagonists flutamide, vinclozolin and procymidone (Gray et al., 1994; Ostby et al., 1999b; Hellwig et al., 2000; McIntyre et al., 2001; Schreiber et al., 2020). It is also noteworthy that finasteride is one of the few identified compounds that can induce 10–12 nipples and shorten AGD to almost female length in male rats without causing other signs of toxicity. Only flutamide, vinclozolin, procymidone and finasteride seem to follow this pattern of complete demasculinization with almost no signs of concomitantly reduced body weights, increased mortality or effects on other target organs such as liver and kidney.

#### Compounds With Clear Effects on NR and No, or Minor, Effects on AGD

Azole fungicides have a somewhat perplexing effect pattern on NR versus AGD. Azole pesticides such as prochloraz, tebuconazole and epoxiconazole all seem to cause a consistent (yet relatively small) increase in NR, but without a significant effect on male AGD. Many azoles seemingly perturb endocrine signaling by disrupting steroidogenesis by inhibiting CYP enzyme activities and can alter steroid hormone concentrations both *in vitro* and *in vivo* as reviewed by (Dreisig et al., 2013).

Prochloraz can inhibit steroidogenesis and antagonize the AR *in vitro* (Vinggaard et al., 2005, 2006). Effects on steroidogenesis are also evident *in vivo* with testosterone output by fetal testis being reduced (Blystone et al., 2007). *In utero* exposure to prochloraz can affect the development of several androgen-sensitive tissues (Vinggaard et al., 2002, 2005; Laier et al., 2006), but it is unclear if these effects are a direct consequence of reduced testosterone levels, antagonistic effects on the AR, or a combination of the two. With the exception of one study reporting shorter male AGD and increased NR at 50 and 150 mg/kg doses (Laier et al., 2006), most studies on prochloraz find no effect on AGD following *in utero* exposure at dose between 25 and 150 mg/kg, yet significant effects on NR (Noriega et al., 2005; Vinggaard et al., 2005; Christiansen et al., 2009; Melching-Kollmuss et al., 2017). This effect pattern is puzzling when viewed against other compounds with similar *in vitro* activities and may be the result of a mechanisms of effect yet to be appreciated or complex *in vivo* toxicokinetics.

Tebuconazole is another azole fungicide that induce NR without a significant effect on male AGD. *In utero* exposure to tebuconazole induces a maximum average effect of 1.6–3.1 retained nipples at 50–100 mg/kg bw/day (Taxvig et al., 2007; Hass et al., 2012), but without a significant shorter AGD. To complicate the matter further, some studies on tebuconazole indicate longer female AGD among littermates exposed to the same doses (Taxvig et al., 2007; Hass et al., 2012).

Epoxiconazole is a third azole fungicide showing an effect pattern where male pups display increased NR but without an effect on AGD. NR in developmentally exposed male pups show a dose-dependent effect pattern and a maximal number of nipples of around 3.4 at doses of 50 mg/kg bw/day and without an effect on male AGD (Taxvig et al., 2007; Hass et al., 2012). As was the case with tebuconazole, epoxiconazole can also induce longer female AGD (Taxvig et al., 2007; Hass et al., 2012). The mode of action for this effect on female AGD is unknown, but could indicate an androgenic mode of action based on our knowledge about hormone-dependent sexual differentiation.

The mild analgesic paracetamol has been suggested to have endocrine disrupting properties (Kristensen et al., 2016). Only one study has investigated the effects on NR (Axelstad et al., 2014). Here, exposure to 360 mg/kg paracetamol induced 30% NR in male offspring but did not affect AGD. By contrast, one study in rats and one study in mice have found that 150 mg/kg paracetamol induced 10–15% shorter AGD in exposed males, but unfortunately NR was not assessed in these studies (Kristensen et al., 2011; Holm et al., 2015), which prevents further comparisons.

Nitrotriazolone (NTO) is a constituent of explosive formulations that, with respect to reproductive toxicity, may fall into the same category as the three above-mentioned azole fungicides. One study has investigated the reproductive toxicity of NTO and find no effects on male AGD, yet small effects on NR at doses from 144 to 3600 mg/L administered in the drinking water (Lent et al., 2016). The mode of action for NTO is unclear as it does not induce marked androgenic or estrogenic effects in the Hershberger and utero trophic assay at doses from 250 to 1000 mg/kg (Quinn et al., 2014).

#### Compounds With No/minor Effects on NR but Effects on AGD

Bisphenol A (BPA) and butyl paraben are both compounds that are considered to be mainly estrogenic in their ED mode of action (Routledge et al., 1998; Kim et al., 2001; Matthews et al., 2001; Byford et al., 2002), albeit they also display anti-androgenic properties *in vitro* (Chen et al., 2007; Rosenmai et al., 2014). Since NR is considered to be caused by an anti-androgenic mode of action, there are not many studies that have assessed NR in rodent toxicity studies with estrogenic chemicals. One study reports a small effect on NR (mean of 0.4 nipples) following exposure to 50 mg/kg BPA (Christiansen et al., 2014). By contrast, four other studies found no effect on NR (Tyl et al., 2002; Howdeshell et al., 2008; Ferguson et al., 2011) even at doses as high as 300–500 mg/kg (Tyl et al., 2002; Delclos et al., 2014), which was also the case with the structural analogue bisphenol C (BPC), with no effects on either NR or AGD at doses of 100 and 200 mg/kg (Gray et al., 2019).

Only one study has investigated if butyl paraben can induce NR and found no effects after exposure to doses of 10–500 mg/kg (Boberg et al., 2016). Interestingly, studies with BPA and butyl paraben have both shown small, but significant, effects on AGD (Christiansen et al., 2014; Boberg et al., 2016), which could suggest that AGD, and not NR, may be regulated by an androgen-estrogen balance, perhaps similarly to what is the case for the genital tubercle which is sensitive to estrogen-androgen balance (Yucel et al., 2003; Yang et al., 2010; Mattiske and Pask 2021). Indeed, studies have shown that the perineal tissues express the estrogen receptor (ER) (Dionne et al., 1979; Schwartz et al., 2019b) and one study shows that polymorphisms in the gene encoding the ER are associated with short AGD in human boys (Sathyanarayana et al., 2012). This might suggest that AGD is a more sensitive endpoint than NR for chemicals with an estrogenic mode of action. However, the much more potent diethylstilbestrol (DES) has been shown to induce some effects on both NR and AGD (Johansson et al., 2021). In this latter study, rats exposed to DES at doses ranging from 0.003 to 0.0012 mg/kg resulted in a mean of 0.4–0.5 retained nipples. Only in the lowest dose group was a small, but statistically significant, effect observed on the male AGD. Thus, further investigations are needed to fully understand the effects of estrogenic compounds on NR and AGD, as well as to explore the possibility of the “estrogenic compounds” also inhibiting androgen production.

#### Compounds With Miscellaneous Effects on NR

We identified 10 additional chemicals that display endocrine modes of action *in vitro* and induce NR in rat offspring. The pattern of effects of these compounds on NR and AGD in developmental toxicity studies were rather variable. It is also worth noting that, for most of them, only one study is available, which complicates the WoE evaluation. For instance, the three pesticides fludioxonil, cyprodinil and dimethomorph have all been shown to have AR antagonistic activity *in vitro;* cyprodinil also showed AR agonistic activity at low concentrations (Orton et al., 2011). When tested *in vivo*, cyprodinil and fludioxonil had no effect on NR at doses between 20 and 180 mg/kg (Scholze et al., 2020). Dimethomorph was tested twice in the same study at doses of 6.7, 20, 60 and 180 mg/kg, with data showing conclusive effects on NR at 180 mg/kg (Scholze et al., 2020).

Ketoconazole is another azole fungicide where both AGD and NR have been assessed in developmental rat toxicity studies. In one study, developmental exposure to 12–50 mg/kg ketoconazole did not induce any effects on AGD or NR (Wolf et al., 1999). In contrast, a second study using doses between 3 and 12 mg/kg observed statistically significant reduced AGD (∼2% at all doses) and increased NR (mean for NR ranging from 0.3 to 1 nipple) in male offspring (Johansson et al., 2021). With the effects in the second study being very small, albeit statistically significant, some caution should be made when interpreting data, especially across studies. However, these clear discrepancies between exposure concentrations and anti-androgenic effects warrant further investigation.

The fungicide mancozeb induced a small, but significant effect on NR at a dose of 25 mg/kg (Hass et al., 2012). The same was seen for perfluorohexane sulfonate (PFHxS) (Ramhøj et al., 2018). The polychlorinated biphenyl (PCB) 126 was tested at a maximal dose of 1 μg/kg, but the NR results were not reported (Rice 1999). One study investigating the effects of the insecticide fenitrothion at doses of ∼1–8 mg/kg finds no effects on NR (Okahashi et al., 2005), while another using doses of 5–25 mg/kg finds 4.1 nipples in male offspring at 25 mg/kg (Turner et al., 2002). It should be noted, however, that in the latter study 20–25 mg/kg also induced maternal toxicity and pup mortality. Similarly, 1.2 nipples were observed in males exposed to 62.5 mg/kg of the cholesterol lowering drug simvastatin, but this was based on only few litters and this dose induced high pup mortality (Beverly et al., 2019).

#### Compounds That Have Only Been Investigated once, Where No Effects Were Found on NR or AGD

Eighteen chemicals had only been investigated in one study, and here no effects were found on NR and AGD. These will not be discussed further here, but can be found in Table 1 and in Supplementary Table S1.

## Modes of Action for Nipple Retention

It is notable that several of the compounds blocking DHT-mediated AR signaling induce a higher number of retained nipples than for instance compounds only reducing testosterone concentrations. These potent anti-androgenic chemicals are finasteride, which blocks the conversion of testosterone to DHT by inhibiting 5-α-reductase, and the three AR antagonists: flutamide, vinclozolin and procymidone. Phthalates, on the other hand, which are known to decrease testosterone levels *in vivo*, tend to cause NR with a smaller effect size (i.e. lower number of nipples per affected male). This alone could suggest that chemicals directly affecting the synthesis of DHT or ligand-activation of the AR have a greater impact on nipple regression than the synthesis of testosterone. Therefore, although testosterone is an essential precursor for DHT production, it seems reasonable to surmise that it is the local concentration of DHT and its ability to activate AR that determines the overall effect on NR in male offspring. The same cause-effect relationship is seen in other tissues, for instance the prostate. Here, testosterone can only partly compensate for the absence of DHT in driving differentiation of the prostate, whereas the absence of both testosterone and DHT completely abolishes prostate differentiation (Imperato-McGinley et al., 1992). However, not all data supports this hypothesis, as some AR antagonists such as linuron and DDE induce a low number of nipples (You et al., 1998; Loeffler and Peterson 1999; McIntyre et al., 2000; Hotchkiss et al., 2004). This is also the case for fludioxonil, cyprodinil and dimethomorph, albeit the cause-and-effect relationship is much less clear for these chemicals as they have only been tested *in vivo* in one study (Scholze et al., 2020).

Phthalates with anti-androgenic properties are often considered to act by inhibiting steroidogenesis which results in reduced fetal testosterone levels *in vivo*. However, the effects caused by phthalate exposure are probably much more complex. *In vitro*, many phthalates appear to cause reduction in testosterone concentrations by activating aromatase with subsequent up-concentration of estrogens, in essence shifting the testosterone/estrogen balance (Lee et al., 2019). Notably, the endocrine disrupting effects of phthalates are not readily predicable from the *in vitro* effects (Lee et al., 2019), which may be due to any number of reasons, including absorption, distribution, metabolism and excretion (ADME), but also the fact that phthalates can cause other adverse effects in cells and tissues at doses where endocrine disruption is observed *in vivo*. For example, phthalates can induce apoptosis of germ cells in mice and rats (Borch et al., 2005; Zhang et al., 2014) and, perhaps more importantly, disrupt Leydig cell integrity in fetal rodent testes (Borch et al., 2005, 2006; Howdeshell et al., 2007; Hannas et al., 2011; Di Lorenzo et al., 2020). Even though the exact mechanism by which phthalates reduce testosterone concentrations *in vivo* remains unclear, the effect of phthalates on NR is most likely mediated by sub-optimal concentrations of testosterone and not by AR antagonism. This may be why most phthalate exposures result in few retained nipples in individual male pups, and only result in some effect on male AGD. In other words, and as discussed previously, reduced testosterone levels in tissues peripheral to the testes may be partially compensated for by local conversion into sufficient levels of DHT. The observation that NR seems to be more responsive to the DHT-AR signaling axis than AGD, however, is more difficult to explain, even if the AGD should be more sensitive to the testosterone-estrogen balance than NR. Regardless, these data suggest that even significantly reduced concentrations of circulating testosterone during the MPW would be enough for the nipples to regress, as it would still provide enough substrate to produce nearly adequate concentrations of DHT in target tissues such as the nipple anlagen. As can be seen in Table 2, the majority of phthalate studies report equal sensitivity of AGD and NR.

**TABLE 2:** NR and AGD as the critical endocrine mediated effect.

	Number of studies that finds the endpoint more sensitive			
Chemical	AGD	NR	Equal	Reference
BBP	1			Tyl et al. (2004)
BPA	1			Christiansen et al. (2014)
Butyl paraben	1			Boberg et al. (2016)
DBP		2	2	Barlow et al. (2004); Lee et al. (2004); Myhlcreest et al. (1999); Mylchreest et al. (2000)
DDE		1	1	You et al. (1998)
DEHP		1	3	Christiansen et al. (2010); Gray et al. (2009); Jarfelt et al. (2005); Moore et al. (2001)
DES			1	Johansson et al. (2021)
DiBP			1	Saillenfait et al. (2008)
DiHP			1	McKee et al. (2006)
DiNP		1		Boberg et al. (2011)
DnHP	1			Saillenfait et al. (2009)
Fenitrothion			1	Turner et al. (2002)
Finasteride	3	1	2	Bowman et al. (2003); Christiansen et al. (2009); Clark et al. (1993); Clark et al. (1990)
Flutamide	1	1	4	Fussell et al. (2015); Hass et al. (2007); Kim et al. (2010); McIntyre et al. (2001); Miyata et al. (2002); Schreiber et al. (2020)
Ketoconazole			1	Johansson et al. (2021)
Linuron		1		McIntyre et al. (2000)
Nitrotriazolone		1		Lent et al. (2016)
Prochloraz		4	1	Christiansen et al. (2009); Hass et al. (2012); Laier et al. (2006); Melching-Kollmuss et al. (2017); Noriega et al. (2005)
Procymidone			2	Hass et al. (2007); Hass et al. (2012)
Pyrifluquinazon		1		Gray et al. (2019)
Tebuconazole		1		Hass et al. (2012))
Vinclozolin		5	5	Christiansen et al. (2009); Flick et al. (2017); Gray et al. (1994); Hass et al. (2007); Hellwig et al. (2000); Matsuura et al. (2005a); Ostby et al. (1999a); Schneider et al. (2011); Wolf et al. (2000)

Number of studies that report on both NR (nipple retention) and AGD (anogenital distance) and find that the LOAEL (lowest observed adverse effect level) is lower for one endpoint than the other or that the LOAEL is similar between the two endpoints. BBP, benzyl butyl phthalate; BPA, bisphenol A; DBP, dibutyl phthalate; DDE, DDT metabolite, dichlorodiphenyl-dichloroethylene; DEHP, diethylhexyl phthalate; DES, diethylstilbestrol; DiBP, di-isobutyl phthalate; DiHP, di-isoheptyl phthalate; DiNP, di-isononyl phthalate; DnHP, di-n-hexyl phthalate.

**FIGURE 2:**
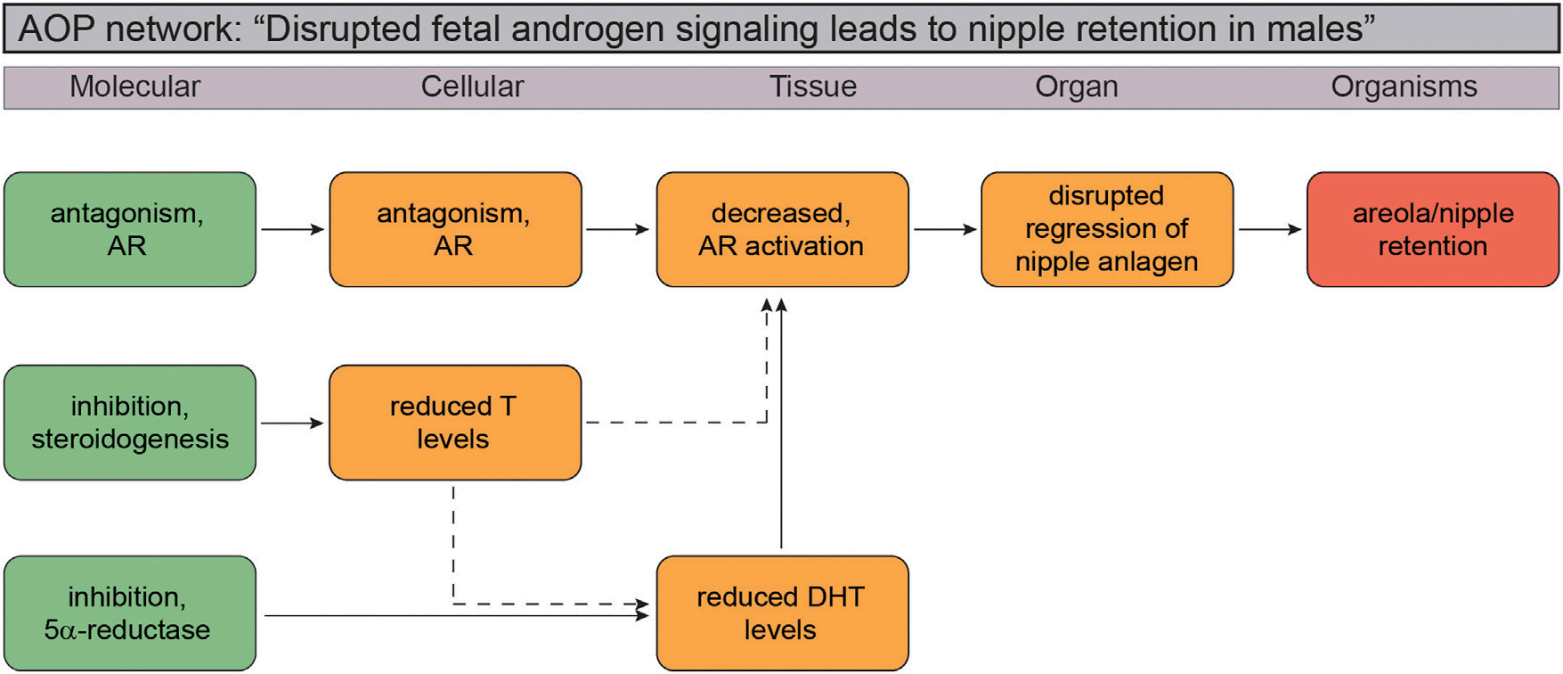
Putative Adverse Outcome Pathway (AOP) network for disrupted androgen signaling leading to nipple retention in male rodent offspring. The small AOP network suggest the molecular initiating event (MIE) can be any of the three vital stages of the androgen signaling axis: testosterone biosynthesis (“inhibition, steroidogenesis”), failure to convert testosterone to dihydrotestosterone (DHT; “inhibition, 5 α -reductase”), or direct antagonism of the androgen receptor (AR; “antagonism, AR”). Any key event that will decrease AR activation and action ultimately “disrupts regression of the nipple anlagen” in male rat offspring, leading to “areola/nipple retention.”

Based on the available literature pertaining to NR in reproductive toxicity studies in rats, and our knowledge about normal reproductive development, it is feasible to elaborate a putative AOP network for NR. This causal pathway relies on the fact that early rodent studies have unraveled the essential role for the DHT-AR signaling axis in preventing nipples from developing in male rats and mice, as well as the fact that available toxicity studies suggest strongest undervirilization effects (NR) with chemical substances that are either potent AR antagonists or prevent the synthesis of DHT. Inhibition of testosterone synthesis itself can also result in NR, but not to the same degree as through the aforementioned mechanisms. Figure 2 depicts a putative adverse outcome pathway (AOP) network for NR in male rat/mouse offspring that we are confident holds the potential to be elaborated into quantitative AOPs in the not so distant future.

It is still not possible to paint a clear picture of a cause-effect relationship from a molecular initiating event to NR, or a “one-size fits all” AOP. Most problematic are incorporation of effect patterns seen with for instance azoles, of which many are known to be potent CYP enzyme inhibitors and thus capable of disrupting steroidogenesis and testosterone production. The challenge is that many of these chemicals also display other mechanisms of action, for example antagonizing the AR. Without even considering the complex issue of ADME of chemicals *in vivo* relative to *in vitro* test assays, multiple mechanisms by single chemicals make it extremely difficult to elaborate naïve cause-effect pathways in complex biological systems. This also extends to the phthalates, where a mechanism of action is assumed rather than confirmed, which makes interpretations of effect data problematic.

## Sensitivity of NR Assessment Compared to That of AGD Measurements

Table 2 summarizes existing toxicological studies that have assessed both AGD and NR in rat toxicity studies. 53 studies had sufficient data to allow for comparison of the lowest dose at which a statistically significant effect was found for each of the two endpoints. For each chemical, studies reporting effects on AGD at a lower dose than NR are listed under the “AGD” column, whereas those reporting an effect on NR at a lower dose than AGD are listed under the “NR” column. Studies where effects were seen at the same dose are grouped under the “Equal” column. Thus, the lowest adverse effect level (LOAEL) was used to determine the sensitivity of NR versus AGD assessment in developmental toxicity studies. As an added note, direct comparisons between the endpoints NR/areolae and AGD is challenged by the fact that the former is more subjective in nature than the latter. We nevertheless find the comparison relevant, and the value will increase in the future with more standardized protocols for NR/areola measurements.

Overall, studies found effects on NR at lower doses than effects on AGD in ∼38% of cases (20/53 studies), AGD was most sensitive in ∼15% of cases (8/53 studies), whereas the two effect endpoints were affected at the same doses in ∼47% of cases (25/53 studies). Taken together, the table shows a varied picture with somewhat limited concordance for each substance. However, as expected, it does identify compounds that generally affect only one of the two endpoints. By reasoning, the endpoint that these compounds affect is more sensitive than the endpoint they do not affect. This includes the azole fungicides tebuconazole and prochloraz which predominantly affect NR, and BPA and butyl paraben which predominantly affect AGD (Table 2). Furthermore, it appears plausible that the four potent anti-androgenic compounds that either antagonize AR (flutamide, procymidone and vinclozolin) or prevent the synthesis of DHT (finasteride) in most cases affect NR and AGD at the same doses. This is the case for flutamide and procymidone whereas the data on vinclozolin and finasteride is more inconclusive. For vinclozolin, some of the disparities can be due to dose choice in the studies, where large spacing between doses makes LOAEL estimations less accurate (*see* underlying data in Supplementary Table S1). Some of the studies investigating the effects of finasteride use doubtful methods to assess AGD and NR or inadequate statistical analysis. For the majority of the remaining compounds the number of studies for each compound is too limited to draw well-founded conclusions.

Taken together, both NR and AGD can provide the critical endocrine mediated effect and give improved understanding of the underlying mechanism. As such, we would like to stress the importance of performing both assessments in order to obtain the most complete information from every study.

## Areola/nipple Retention - A Permanent or Transient Effect in Rats?

There is an ongoing debate whether or not NR measured in 2-week old rat offspring is a transient or a permanent effect. Some rat toxicity studies have reported on NR at PND12/13, but with retained nipples no longer visible a week later (Melching-Kollmuss et al., 2017). However, a sizeable number of other studies suggest NR to be permanent. This distinction between a transient and a permanent effect is important from a regulatory perspective, since only a permanent effect will be categorized as a malformation according to an OECD guidance document (OECD 2008).

Of the 147 studies included in this review, 65 have assessed NR later than PND 11–18. Out of these 65 studies, 14 studies did not see NR in infant males and were thus not included in our overall evaluation of whether NR is a permanent or a transient effect. For this analyses, the remaining 51 studies were included and are listed in Table 3.

**TABLE 3:** Nipple retention (NR) as a permanent or transient effect.

Substance	Permanent	Transient	Reference
BBP	2	0	Gray et al. (2000); Hotchkiss et al. (2004)
DBP	6	2	Barlow et al. (2004); Carruthers and Foster (2005); Clewell et al. (2013); Saillenfait et al. (2008); Wolf et al. (1999)
DDE (p,p'-DDE)	2	0	Wolf et al. (1999)
DEHP	4	0	Gray et al. (2000); Saillenfait et al. (2009); Wolf et al. (1999); Moore et al. (2001)
DiBP	1	0	Saillenfait et al. (2008)
DINP	1	0	Gray et al. (2000)
DnHP	1	0	Saillenfait et al. (2009)
Fenitrothion	0	1	Turner et al. (2002)
Finasteride	3	3	Bowman et al. (2003); Imperato-McGinley et al. (1992); Martínez et al. (2011); Clark et al. (1990)
Flutamide	6	1	Imperato-McGinley et al. (1992); Foster and Harris (2005); Fussell et al. (2015); McIntyre et al. (2001); Miyata et al. (2002)
Linuron	3	0	Hotchkiss et al. (2004); McIntyre et al. (2002); Wolf et al. (1999)
Prochloraz	1	1	Noriega et al. (2005); Melching-Kollmuss et al. (2017)
Procymidone	2	0	Ostby et al. (1999b); Wolf et al. (1999)
Simvastatin	0	1	Beverly et al. (2019)
Vinclozolin	9	1	Hellwig et al. (2000); Ostby et al. (1999a); Schneider et al. (2011); Wolf et al. (2000); Wolf et al. (2004); Flick et al. (2017)

Number of studies that report whether the effect on NR is permanent or transient. BBP, benzyl butyl phthalate; DBP, dibutyl phthalate; DDE, DDT metabolite, dichlorodiphenyl-dichloroethylene; DEHP, diethylhexyl phthalate; DiBP, di-isobutyl phthalate; DiNP, di-isononyl phthalate; DnHP, di-n-hexyl phthalate.

As evident from Table 3, the majority of studies support the view that NR is generally a permanent effect, with 41 out of 51 studies (∼80%) reporting permanent nipples in mature male rats. Only 10 out of the 51 studies (∼20%) found no effect at the later time point (Supplementary Table S2). Notably, several studies deal with the same chemical, but there are still 15 different substances representing various mechanisms of action. Again, the potent anti-androgens flutamide, vinclozolin and procymidone generally show permanent effects (17 of 19 studies). One study on flutamide finds transient NR but uses a short, and late (GD18), exposure window (Foster and Harris 2005), which suggests that the androgen-dependent regression of the nipples is almost completed before GD18 in rats and not that the effect is transient. In the single study of vinclozolin that does not find permanent NR it is not specified if the male offspring were shaved before assessment (Flick et al., 2017). If they were not, this would likely have impaired the ability to assess permanent nipples, as shaving the abdomen is usually necessary in order to visually observe NR in mature rats.

Even for the phthalates (where the frequency of male pups presenting with NR is relatively high but the number of retained nipples are relatively low in each individual pup) the vast majority of studies report on permanent nipples. This includes BBP (two of two studies), DBP/DiBP (seven of nine studies), DEHP (four of four studies), DiNP (one of one study) and DnHP (one of one study). With regard to the DBP studies reporting transient NR, it is notable that permanent nipples were observed after exposure on GD16 and GD17, but not after exposure on either GD15-16 or GD17-18 (Carruthers and Foster 2005). Thus, as previously mentioned for flutamide, this lack of “permanent effect” may come down to exposure period. After exposure on GD15-16, around 3% of the males were found to have permanent nipples, albeit not statistically significant, suggesting that the main window of sensitivity for phthalate-induced permanent NR is GD16-17. For most reproductive toxicity studies, however, the exposure period spans the critical window for when nipples should normally regress in males.

There are several other compounds with anti-androgenic properties that induce permanent nipples in rats following *in utero* exposure. This includes linuron (Wolf et al., 1999; McIntyre et al., 2001; Hotchkiss et al., 2004), DDE (Wolf et al., 1999), and prochloraz (Noriega et al., 2005). Prochloraz has also been reported to cause only transient NR (Melching-Kollmuss et al., 2017). However, the study reporting transient NR has limited sensitivity as the males were seemingly not shaved before assessment. The AR antagonist fenitrothion did not induce permanent nipples (Turner et al., 2002). The 5α-reductase inhibitor finasteride has induced both permanent and transient NR (Clark et al., 1990; Imperato-McGinley et al., 1992; Bowman et al., 2003; Martínez et al., 2011). Notably, three out of six finasteride studies do not find permanent nipples, but different exposure periods and dose levels were used in these three *in vivo* studies which makes comparisons difficult (Clark et al., 1990).

Taken together, the majority of studies support that NR is a permanent effect. The data also highlights that the validity of NR data is dependent on proper measurement and recording, which will be discussed in more detail in the following sections.

## NR in Regulatory Toxicology and Recommendations for Proper Measurements in Rodent Studies

### NR in International Test Guidelines

The ectopic expression of nipples/areolae in infant male offspring is included as a mandatory endpoint in several OECD test guidelines (TGs) for development and reproductive toxicity. NR is assessed in all offspring (male and female) at PND 12–14 under the two screening studies for reproductive toxicity (TG 421 and TG 422) (OECD 2016a, b), as well the extended one-generation study (TG 443) (OECD 2018). NR is, however, not included in the test guideline for the current two-generation study (TG 416) (OECD 2001), but requested by EU member states as a default endpoint when the two-generation study is conducted instead of the OECD TG 443 study to address endocrine evaluations (EFSA, 2020). NR can be used in chemical risk assessment to set the NOAEL as stated in the OECD guidance document 151, which guides the interpretation of TG 443 (OECD 2013): “*A statistically significant change in nipple retention should be evaluated similarly to an effect on AGD as both endpoints indicate an adverse effect of exposure and should be considered in setting a NOAEL*.”

Assessment of permanent nipples in mature males is not specifically mandated in any OECD TG, but only suggested indirectly in that abnormalities should be recorded at necropsy. It is our distinct impression that permanent nipples are rarely, if ever, recorded in TGs studies. Although we are not advocating that adult NR should be mandated in all studies, we hope that our discussion can promote the recording of NR in mature males when relevant in future studies and thereby help us improve on our knowledge pertaining to NR for the sake of improving its utility as a continued *in vivo* biomarker.

### Practical Considerations for Assessing NR in Rats

Assessment of NR in male rat pups can be highly subjective since it involves the detection of relatively small, dark spots located along the “milk lines” in positions corresponding to where the female nipples are located (Figure 4). As previously mentioned, at early developmental stages they are only pigmented patches (areolas) rather than actual nipples. Consequently, the number of nipples that are recorded–including in control males–may be influenced by factors such as age of the pups at assessment, the quality of the light source, and the experience of the assessor.

When examining NR, the areolas/nipples could be reported either as a qualitative (binominal) yes/no answer or quantitatively by counting the number of nipples. In OECD GD 151 (OECD 2013) it is recommended to provide the quantitative count as a qualitative assessment gives a low statistical power. A qualitative assessment may therefore be rather insensitive, particularly when the incidence of retained nipples in control animals is high.

The presence of nipples/areolas must be assessed at correct developmental stages. Areolas are only visible on the skin and therefore not identifiable as the pups start to grow fur. On the other hand, assessing nipples/areolas at early time points, for instance at PND4, is not feasible since the nipples are not developed enough to be identified (OECD 2015). As a result, OECD TGs stipulate that NR should be assessed in males when nipples are visible in their female littermates; that is, at PND12-14 depending on the rat strain. When assessing more mature pups for permanent nipples, it is important to shave the abdomen of the animals as fur makes it difficult, if not impossible, to properly detect the presence of nipples at this age, especially if they present as areolas rather than fully developed nipples. Importantly, all pups within the same study should be evaluated on the same days to account for differences in maturation.

In addition, actual “criteria” for a positive score may differ between labs and assessors, a fact that can make direct comparisons between studies problematic. This is also why, nipples/areolas should ideally be assessed by the same assessor for each study, and this person(s) should be blinded to exposure group. Swapping between assessors or knowing the exposure group of the investigated offspring could inadvertently skew the data. For CROs performing guideline studies for developmental and reproductive toxicity these criteria may be challenging or even impossible to adhere to, due to logistical considerations. When this is the case, we recommend that the performing laboratories arrange training sessions for the technical personnel, in order to aligning their assessments as much as possible. The same issue extends to AGD measurements, where it is critical that measurement are performed the same way for each animal to avoid “technical variations” that could obscure subtle changes between study groups.

### NR in Control Animals

It would be easy to assume that male control animals always have 0 (zero) nipples. It is, however, not that simple. Retained areolas/nipples are sometimes observed in control male rats and assessment of NR in control animals (as well as knowledge of the number of nipples one can expect to find in control animals) is imperative for assay validity and accurate interpretation of NR data from toxicological studies. In other words, the establishment of a reliable number of areolas/nipples in control animals is important for the detection of substance-induced effects.

An example of how this can be an issue can be gleaned from a large generation study on oxybenzone published by NTP in US (https://tools.niehs.nih.gov/cebs3/views/?action=main.dataReview&bin_id=14485). Here, close to nine hundred male rats were assessed for NR at PND 13. Despite the large number of animals, no nipples were seemingly found in any of the pups from either control or exposed groups. This finding is surprising, since some degree of NR is expected even in control males. This result–zero areolas/nipples in control males–brings into question whether the assessment of NR was performed in accordance with good practices or if “good practices” for measuring NR in infant male rats is in itself sufficiently standardized. To further investigate the “expected background NR rate,” we analyzed an extensive database with NR data from our own animal experiments performed between 2003 and 2019. Based on 35 control groups (Table 4), we find that the control values are 0 in only three out of 35 studies (8.6%), suggesting that control values of 0% are uncommon. It should be noted that these studies were mainly performed with Wistar rats, but in several recent studies with Sprague-Dawley rats. Comparison of the control values for these two rat strains commonly used in toxicological tests shows quite similar values for both mean number of NR and for % pups with NR (Table 4).

**TABLE 4:** Control values for nipple retention (NR).

Strain	Studies	Litters	No males	No., mean ± Std	% pups NR, mean	% control groups with NR = 0.00
Both	35	422	2165	0.26 ± 0.44	14.0	8.6
Wistar	28	349	1729	0.27 ± 0.49	13.1	11.5
SD	7	73	436	0.22 ± 0.15	17.4	0.0

Control values for NR in our own studies, performed between 2003 and 2019. Studies in both Wistar and Sprague Dawley rats are included. In total, data from 35 studies are included. NR, nipple retention; No., number; SD, Sprague Dawley; Std, standard deviation.

**FIGURE 3:**
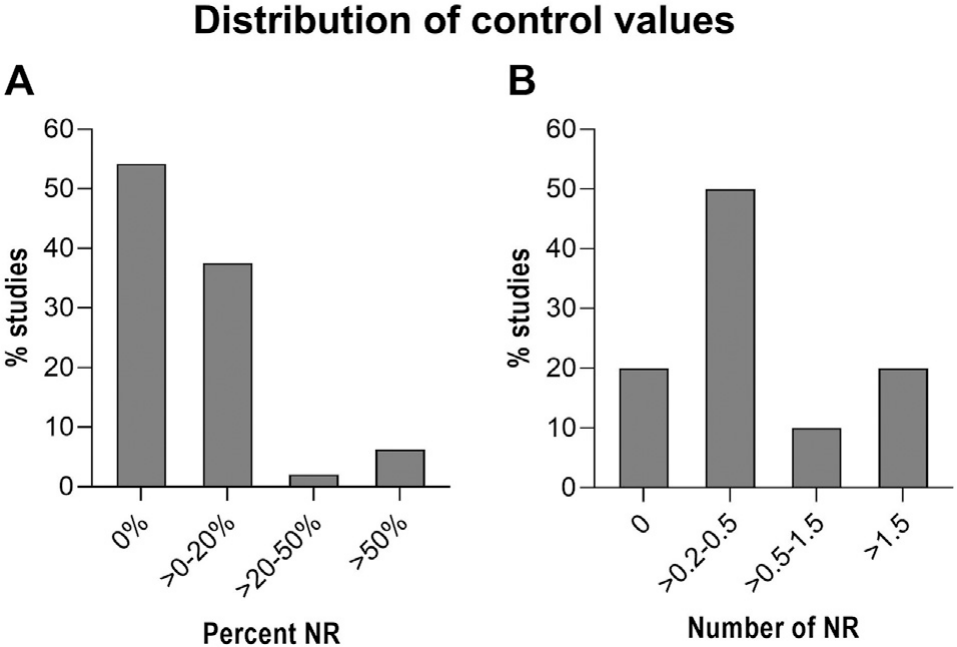
Distribution of Nipple retention (NR) in control rats/groups. Control rats/groups from the 80 studies in Supplementary Table S1. **(A)**: Results shown as percent NR in the control groups. **(B)**: Results shown as number of nipples in the control groups.

To further substantiate our concern, we also examined all of the reported control levels in the studies shown in Supplementary Table S1, giving a total of 80 male control groups. Out of the 80 control groups, 54% report a control value of 0% (Figure 3A). Again, we find this to be surprisingly high based on personal experience. The picture becomes somewhat different when control values are reported as a number rather than as percentage (Figure 3B). In this case, 20% of 43 studies show zero nipples in male controls. This difference could be coincidental, but might also indicate that personnel trained to *count* NR are superior at detecting NR than those who only score a yes/no response.

Studies reporting very high control levels may also raise concern as this could mask any treatment related effects. A total of 48 studies reported the control values as % positives (i.e. as a yes/no answer). The majority of these studies (∼92%) report values below 20% (Figure 3A). Only three out of the 48 studies report control values above 50% (Fussell et al., 2015; Flick et al., 2017; Melching-Kollmuss et al., 2017). These three studies on prochloraz, vinclozolin and flutamide are from the same industry group and report control values of 65–67%, which are unusually high for control groups. This calls into question the sensitivity of the testing. In such cases we recommend to scrutinize the criteria used for scoring positives or for counting areolas/nipples. For instance, the prochloraz study reports an increase in NR at PND 12 and the authors argued that “*the historical background incidence of retained nipples/areola is high at the observation time point PND 12 and is observed in percentages up to 70% in untreated controls, raising some doubts on the treatment relationship of this finding*” (Melching-Kollmuss et al., 2017). Notably, a similar argument is not used in the studies on flutamide and vinclozolin, where a similarly and unusually high control values are reported (Fussell et al., 2015; Flick et al., 2017).

Overall, given both the published data (Figure 3) and the calculations based on our own raw data (Table 4), a control value around 10–20% or a nipple count ranging from larger than zero to around 0.5 appears to be most common for control males. This raises concern for the validity of larger studies reporting a control value of 0% or 0.0; especially in cases where this is seen in several studies and where no exposure-related effects are found. In such cases, we recommend to perform a positive control study focusing on doses inducing low, but significant NR/areolas. A more comprehensive overview of historical control data could be achieved by including unpublished data from CRO/Industry for this endpoint, routinely collected in OECD TG 443, 421 and 422 studies.

## Supernumerary Nipples in Humans–Any Relevance to Endocrine Disruption?

**FIGURE 4:**
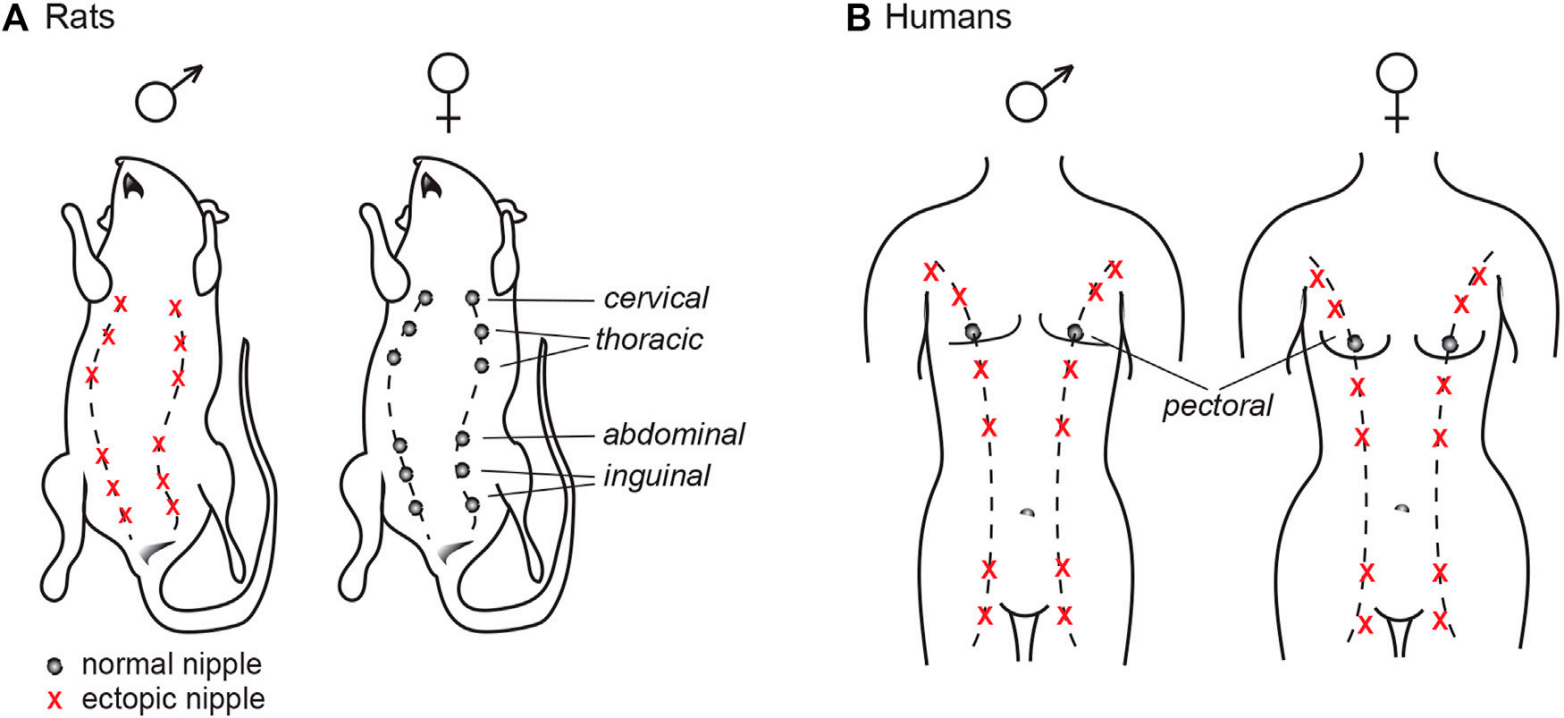
The mammalian milk line, or mammary ridge. **(A)** In common laboratory rats (such as Wistar and Sprague Dawley), nipples will only develop in the female offspring, as androgen signaling during development prompts the nipple anlagen to regress in males. **(B)** Humans have auxiliary mammary ridges, but only the two central pectoral nipples will develop. Contrary to rats, both sexes have the same number of nipples.

Just like other mammals, humans possess a rudimentary mammary ridge, or “milk lines,” running along the front torso from the arm pits (axilla) to the upper medial side of the thighs (Figure 4). They first appear between gestational week 4 and 5, but will regress in a caudal to cranial direction later in development (Leung and Robson 1989). Humans typically have two nipples, regardless of sex, that are located at the fourth rib and will form the pectoral breasts. In some individuals, regression of the nipple anlagen is not complete which may results in supernumerary (ectopic) nipples, or polythelia. A single extra nipple is most common, but multiple extra nipples may also occur. The “third nipple” is most frequently located along the mammary ridges and often present between the pectoral nipple and the naval (umbilicus); albeit, excess nipples can appear in other places such as face, neck, vulva and thighs (Leung and Robson 1989; Brown and Schwartz 2003). Supernumerary nipples are surprisingly common and occur in 0.2–6% of the population, with prevalence varying greatly amongst geographic regions and ethnicities (Mimouni et al., 1983; Leung and Robson 1989; Brown and Schwartz 2003). The etiology of supernumerary nipples, however, remains poorly characterized.

There are familial cases of supernumerary nipples displaying a pattern of autosomal dominant transmission with variable penetrance, but most cases appear to be sporadic. Thus, although genetics is suspected to play a dominant role in the etiology of excess nipples in humans, the responsible genes remain elusive. Interestingly, supernumerary nipples have been associated with numerous diseases, most prominently urogenital disorders (Méhes et al., 1987; Leung and Robson 1989; Grotto et al., 2001; Brown and Schwartz 2003). This supports the view that genetics is a major factor, but also allows for the possibility of environmental influences. Whether or not endocrine disruption play a significant role, however, cannot be determined as there is almost a complete lack of epidemiological studies looking at associations between excess nipples and either altered hormone profiles or exposure to EDCs. Since males and females express the same number of nipples in humans, the influence of androgens would necessarily be different than what is the case in rats. However, this does not exclude the possibility that hormone disruption could play some part. At least sex hormones are major regulators of nipple/breast development at puberty, a developmental stage when diffuse areolas/moles in humans can develop into nipples and breast tissues (Mimouni et al., 1983; Leung and Robson 1989). Nevertheless, until we have data available, a link between endocrine disruption and supernumerary nipples in humans remains speculative; but it would certainly be an interesting issue to explore in future studies.

## Conclusions and Perspectives

The retention of areolas/nipples in male rodent offspring after *in utero* exposure to chemicals is a sign of perturbed androgen action during critical stages of the “masculinization programming window.” As adverse outcomes that can be observed in intact animals (as currently required to categorize a chemical substance as an EDC from a regulatory point of view), both NR and AGD are essentially markers for the same mode of action: anti-androgenic. In this regard, measuring both endpoints could be considered redundant. However, as our current synthesis of available data has shown, there are differences in specificity and sensitivity between the two biomarkers. In addition, these differences may stem largely from the fact that the molecular pathways governing development of the perineal region and the nipple anlagen in rats are different. Both processes are highly sensitive to androgen action, but there are data also strongly suggesting that AGD is influenced by additional mechanisms/modalities outside the linear, and canonical, testosterone-DHT-AR signaling pathway in a way that nipple regression is not.

To summarize, we want to highlight the following five points to stress why we think NR is of continued value as a biomarker of anti-androgenicity, as well as some points that should be of focus in the future.

- NR is a sensitive endpoint for anti-androgenic effects in developmental/reproductive rodent toxicity studies and is relevant for human risk assessment purposes
- In most cases, NR and AGD reveals similar effect data as pertaining to endocrine disruption through anti-androgenic mode of action, but in some cases NR is more sensitive than AGD. The assessment of both NR and AGD in guideline studies is therefore still recommended
- We recommend an international standardization to reduce the lab-to-lab variability in the outcome of NR assessments
- NR may be more specific than AGD with regard to anti-androgenic mode of action (specific to the DHT-AR signaling axis), with AGD seemingly also more vulnerable to perturbations outside the canonical DHT-AR signaling axis; e.g. substances with estrogenic action
- Most data indicate that NR is a permanent effect
